# Longitudinal associations between PTSD symptom clusters and domains of hazardous drinking among a cohort of United States Army Reserve and National Guard soldiers

**DOI:** 10.1111/acer.70211

**Published:** 2026-01-23

**Authors:** Rachel A. Hoopsick, Malaiikha McCormick‐Cisse, D. Lynn Homish, Gregory G. Homish

**Affiliations:** ^1^ Department of Health and Kinesiology University of Illinois Urbana‐Champaign Champaign Illinois USA; ^2^ Department of Community Health and Health Behavior University at Buffalo Buffalo New York USA

**Keywords:** alcohol‐related problems, consumption, dependence, military, PTSD

## Abstract

**Background:**

Posttraumatic stress disorder (PTSD) and hazardous drinking remain significant problems in military‐connected populations. Prior research suggests that specific PTSD symptom clusters may relate differently to hazardous drinking behaviors, but longitudinal data are limited, especially among reservists.

**Methods:**

Using a subset of data (*N* = 485 US Army Reserve/National Guard soldiers) from the Operation: SAFETY study, we examined the longitudinal associations between PTSD symptom clusters (re‐experiencing, avoidance, negative cognitions and mood, hyperarousal) and domains of hazardous drinking (consumption, dependence, alcohol‐related problems) measured by the AUDIT. We used generalized estimating equation models adjusting for anxiety and depression, and interactions with biological sex were tested.

**Results:**

Controlling for the effects of time and anxiety and depression symptomatology, re‐experiencing symptoms were not associated with any domains of hazardous drinking (*p*s >0.05). Greater avoidance symptoms (*p* < 0.001) and negative cognitions and mood (*p* < 0.05) were both associated with greater alcohol‐related problems over time. Greater symptoms of hyperarousal were associated with greater consumption (*p* < 0.05) and alcohol‐related problems (*p* < 0.01). No symptom clusters were associated with dependence (*p*s >0.05). There was a significant interaction between symptoms of hyperarousal and sex on alcohol‐related problems (*p* < 0.01), such that greater symptoms of hyperarousal were associated with greater alcohol‐related problems over time among male soldiers, but not female soldiers.

**Conclusions:**

Findings suggest that specific PTSD symptom clusters, rather than overall severity, may influence hazardous drinking. These effects primarily manifest in consumption behaviors and alcohol‐related problems, with greater risk observed among male soldiers experiencing hyperarousal features of PTSD.

## Graphical abstract

Among Army Reserve and National Guard soldiers, PTSD symptom clusters, particularly hyperarousal, avoidance, and negative cognitions and mood, were linked to hazardous drinking over time. Findings reveal symptom‐specific pathways between trauma and alcohol use that inform prevention and conceptual models of comorbidity in military populations.

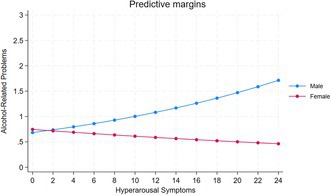

## INTRODUCTION

Research suggests that the prevalence of alcohol misuse among those with posttraumatic stress disorder (PTSD) and the prevalence of PTSD among those with alcohol misuse may both exceed 60% (Debell et al., 2014). This high rate of comorbidity suggests a complex interplay between trauma exposure and alcohol misuse (Banducci et al., 2019; Green et al., 2014; Smith & Cottler, 2018; Weiss et al., 2020), and both are more prevalent among military populations as compared to their civilian counterparts (Smith & Cottler, 2018). Military service often involves exposure to combat, witnessing death, and experiencing life‐threatening situations, all of which significantly increase the risk for PTSD (Xue et al., 2015). Simultaneously, the cultural norms within military settings, which often emphasize alcohol use as a coping mechanism or a means of social bonding, further contribute to the high rates of hazardous drinking observed in this population (Jones & Fear, 2011; Meadows et al., 2023). Understanding the nuanced relations between specific PTSD symptom clusters and features of hazardous drinking may be important for developing targeted interventions that effectively address these interrelated phenomena. However, this area remains underexamined in military populations who are at elevated risk for both PTSD and alcohol misuse, particularly among military reservists (Cohen et al., 2015).

Although the link between overall PTSD severity and hazardous drinking has been well established in various populations (e.g., Bartlett et al., 2019; Tripp et al., 2020; Wolitzky‐Taylor et al., 2023), the clinical presentation of PTSD can vary widely (Friedman et al., 2011). PTSD can be characterized across four primary symptom clusters: re‐experiencing, avoidance, negative cognitions and mood, and hyperarousal (American Psychiatric Association, 2013). Re‐experiencing (cluster B) symptoms involve intrusive thoughts, nightmares, and flashbacks related to the traumatic event. Avoidance (cluster C) symptoms entail efforts to evade reminders of the trauma, which can lead to social isolation and emotional numbing. Negative cognitions and mood (cluster D) symptoms encompass persistent negative beliefs, diminished interest in activities, and feelings of detachment. Hyperarousal (cluster E) symptoms include heightened irritability, hypervigilance, and sleep disturbances. Each of these clusters may differentially influence alcohol‐related behaviors and outcomes, and there is a need for a more nuanced examination of the relations between PTSD symptom clusters and hazardous drinking (Walton et al., 2018).

Multiple theoretical frameworks support the associations between PTSD and alcohol misuse in military‐connected populations, including both the self‐medication hypothesis and the negative reinforcement model (Baker et al., 2004; Khantzian, 1997). There is significant evidence to support the self‐medication hypothesis, in which military service members drink alcohol to cope with PTSD, especially symptoms of hyperarousal like insomnia, irritability, and anxiety (Hawn et al., 2020; Luciano et al., 2022). However, the directionality of the association between PTSD and alcohol use is not entirely understood. There is growing support for the negative reinforcement model (Baker et al., 2004), which purports that service members often drink to reduce distress rather than for social pleasure, and that alcohol use temporarily relieves PTSD symptoms thereby reinforcing the drinking behavior over time. For example, Jakupcak et al. (2010) found that emotional numbing symptoms were most strongly associated with alcohol misuse among Iraq and Afghanistan war veterans, suggesting that drinking may serve as an avoidant coping strategy, which may represent a critical pathway maintaining comorbid PTSD and alcohol problems in veteran populations.

However, there are often significant differences in the presentation of PTSD and downstream sequelae, including alcohol misuse, by biological sex (Christiansen & Berke, 2020; Christiansen & Elklit, 2012; Olff, 2017; Tolin & Foa, 2008). Women often report higher levels of avoidance and negative cognitions/mood, while men may exhibit higher hyperarousal and externalizing symptoms (Christiansen & Elklit, 2012). These differences extend to alcohol use: Men generally report higher consumption and are more likely to use alcohol as a coping strategy, whereas women may engage in more internalizing patterns of distress (Keyes et al., 2012). Understanding sex‐specific patterns is critical for tailoring interventions and interpreting associations between PTSD symptom clusters and hazardous drinking in military reservists.

One cross‐sectional study of 100 veterans seeking outpatient treatment for both PTSD and substance use disorders found that re‐experiencing symptoms, negative cognitions and mood symptoms, and hyperarousal symptoms were each associated with greater alcohol misuse (Walton et al., 2018). However, this was a small and clinical sample, and even at subthreshold levels, symptoms of PTSD can cause substantial distress and adversely affect health and well‐being (Homish et al., 2019; Marshall et al., 2001; Zlotnick et al., 2002). A more robust study of 1295 veterans demonstrated that specific symptoms of PTSD were associated with future drinking behaviors (Livingston et al., 2022). In this longitudinal study by Livingston et al. (2022), the hyperarousal features of PTSD predicted future binge drinking, and avoidance of internal stimuli was an important differentiator of those at high and low risk for alcohol problems. However, this study did not examine any differences in these relations by sex, and the authors noted that additional studies that examine specific PTSD symptom clusters (rather than global PTSD symptom severity) are needed to maximize research and clinical impact (Livingston et al., 2022).

Less is known about PTSD and hazardous drinking among a specific high‐risk military subpopulation: reservists. These part‐time military service members, comprising more than one‐third of the US military, have been shown to be at a greater risk for experiencing problems with both mental health and substance use compared to their active‐duty counterparts (Cohen et al., 2015; Jacobson et al., 2008, 2020; Milliken et al., 2007; Wang et al., 2020). Preliminary cross‐sectional research in a community sample of reservists shows that both greater overall PTSD severity and greater individual symptom cluster (B, C, D, and E) severity are associated with more hazardous drinking among reservists (Homish et al., 2019). However, it remains unclear which domains of hazardous drinking (i.e., consumption, dependence, and alcohol‐related problems) as measured by the Alcohol Use Disorders Identification Test (AUDIT) might be affected. These relations might otherwise be obscured when examining overall PTSD symptom severity or total AUDIT scores alone. Moreover, it is not known if these associations persist over time or differ by sex.

This study addresses the following exploratory research questions: (1) Which PTSD symptom clusters are associated with specific domains of hazardous drinking over time? and (2) do these associations differ by biological sex? By examining PTSD symptom clusters and domains of hazardous drinking over time, rather than overall PTSD symptom severity and total AUDIT scores cross‐sectionally, this study aims to provide a deeper understanding of how these potentially nuanced associations and interactions evolve and persist over time. This knowledge might inform case conceptualizations and help to identify soldiers at elevated risk.

## MATERIALS AND METHODS

### Participants and procedure

We recruited participants for the Operation: SAFETY (Soldiers and Families Excelling Through the Years) study from 47 USAR/NG units across the state of New York over a 15‐month period between the summer of 2014 and the fall of 2015. Operation: SAFETY was described as a study of how soldiers and spouses impact each other's physical and mental well‐being. These participants consisted of adult couples who were married or living as if married at baseline, in which at least one member of the dyad was a current USAR/NG soldier. Eligible couples completed electronic surveys annually after giving informed consent to participate, with follow‐up surveys administered approximately 1 year after each participant's prior survey date. Data were collected using a secure, web‐based survey platform. Participants accessed these web‐based assessments via a unique, individualized username and password on a computer at the University at Buffalo or on a personal device at another place of their choosing. All responses were encrypted and stored on a secure, access‐restricted university server. Each participant received a check for $60 at baseline, $70 for each of the first two follow‐up surveys (i.e., year 1, year 2), $80 for the third follow‐up (i.e., year 3), and $90 for the fourth follow‐up (i.e., year 4). The study protocol was approved by the Institutional Review Board at the University at Buffalo, the Army Human Research Protections Office, the Office of the Chief, Army Reserve, and the Adjutant General of the National Guard.

A total of 731 soldiers and partners were eligible for inclusion in Operation: SAFETY. Of those, 572 (78%) agreed to participate, and 83% of these couples (*N* = 472 couples) completed some part of the survey. Surveys were included only if both partners completed follow‐up (*N* = 418 couples). Of the 418 couples enrolled at baseline for the Operation: SAFETY study, only 25 couples (6.0%) did not participate in the year 4 follow‐up survey (both partners were lost to follow up). We conducted sensitivity analyses and found that if a civilian partner screened for the study (*n* = 11 couples), the couple was less likely to enroll (*p* < 0.001) than if a soldier screened for the study. The analytic sample for the current study includes 485 male and female USAR/NG soldiers. Soldiers were predominantly non‐Hispanic White, had at least some college education, and were in their early 30s at baseline. Additional sample characteristics are presented by biological sex in Table 1.

**TABLE 1:** Participant characteristics at baseline by biological sex (*N* = 485).

	Male soldiers (*n* = 387), mean (SD) or % (*n*)	Female soldiers (*n* = 98), mean (SD) or % (*n*)
Age, years	31.7 (6.6)	30.5 (6.1)
Race/ethnicity		
Non‐Hispanic White	79.3% (307)	82.7% (81)
Non‐Hispanic Black	5.9% (23)	2.0% (2)
Hispanic	9.0% (35)	7.1% (7)
Other	4.1% (16)	6.1% (6)
Education		
High school	15.0% (58)	5.1% (5)
Some college	56.6% (219)	55.1% (54)
College degree	28.4% (110)	39.8% (39)
Family income		
Less than $20,000	6.2% (24)	13.3% (13)
$20,000 to $39,999	20.2% (78)	19.4% (19)
$40,000 to $59,999	22.7% (88)	20.4% (20)
$60,000 to $79,999	19.4% (75)	17.4% (17)
$80,000 to $99,999	11.6% (45)	11.2% (11)
$100,000 to $119,999	8.3% (32)	5.1% (5)
$120,000 or more	8.8% (34)	9.2% (9)
Years of military service	9.7 (6.1)	7.0 (4.7)
Rank		
Enlisted	84.2% (326)	79.6% (78)
Officer	14.2% (55)	11.2% (11)

Abbreviation: SD, standard deviation.

### Measures

#### PTSD

We assessed participants' PTSD symptomatology using the 20‐item Posttraumatic Stress Disorder Checklist (PCL‐5; Bovin et al., 2016, Weathers et al., 2013). The PCL‐5 examines how much respondents are affected by each of the 20 symptoms of PTSD in the *Diagnostic and Statistical Manual of Mental Disorders, Fifth Edition (DSM‐5)* across four symptom clusters (i.e., cluster B: re‐experiencing, cluster C: avoidance, cluster D: negative cognitions and mood, cluster E: hyperarousal) over the past month. Each item is scored on a Likert scale ranging from 0 (not at all) to 4 (extremely). Individual items are summed to create a total score ranging from 0 to 80, with higher scores indicating greater posttraumatic stress (*α* = 0.95). Re‐experiencing the event (cluster B) consists of five items (0–20) covering memories, dreams, flashbacks, and physical reaction (*α* = 0.92). Avoidance (cluster C) is made up of two items (0–8) for avoiding internal and external memories (*α* = 0.89). Negative mood (cluster D) is comprised of seven items (0–28) and examines dissociative amnesia, negative beliefs/feelings, blame, negative affect, detachment or estrangement, and numbing (*α* = 0.91). Lastly, hyperarousal (cluster E) consists of six items (0–24) covering irritability/aggressive behavior, reckless behavior, hypervigilance, startle, concentration, and sleep (*α* = 0.84). All PTSD symptom clusters were entered as count variables in our analyses.

#### Hazardous drinking

We used the Alcohol Use Disorders Identification Test (AUDIT) to assess hazardous drinking and its domains (Babor & Del Boca, 1992; Saunders et al., 1993). This 10‐item measure consists of questions about current alcohol consumption, symptoms of alcohol dependence, and alcohol‐related problems. Questions are scored 0–4 on a Likert scale with responses ranging from “Never” to “Daily or Almost Daily.” Summary scores range from 0 to 40, with higher scores indicating greater alcohol problems. The AUDIT had good internal consistency in our sample (*α* = 0.78). Total AUDIT and subscale (i.e., alcohol consumption [3 items], alcohol dependence [3 items], alcohol‐related problems [4 items]) scores were entered as count variables in all analyses.

#### Sex

All participants self‐reported their biological sex (i.e., male, female) at the baseline assessment, and it was treated as a binary variable in our interaction models.

#### Anxiety

We used the Severity Measure for Generalized Anxiety Disorder (Craske et al., 2013) to assess participants' symptoms of anxiety over the past week. This 10‐item measure has high internal consistency (*α* = 0.91) and maps onto the *DSM‐5*. Example items include feeling “moments of sudden terror, fear, or fright,” feeling “anxious, worried, or nervous,” and feeling “a racing heart, sweaty, trouble breathing, faint, or shaky.” Items are scored on a 5‐point Likert scale ranging from 0 (never) to 4 (all of the time), and individual item scores are summed to create a total score (range: 0–40), with higher scores indicating greater anxiety symptomatology. Total anxiety score was entered as a count variable in adjusted models.

#### Depression

We assessed participants' depression symptomatology with the 8‐item Patient Health Questionnaire (PHQ‐8; Kroenke et al., 2009), a modified version of the 9‐item Patient Health Questionnaire (PHQ‐9; Kroenke et al., 2001), which omits an item on suicidal thoughts. This measure assesses the frequency with which the respondent has been affected by depressed states over the last 2 weeks and includes example items such as “Feeling down, depressed, or hopeless” and “Feeling bad about yourself.” The PHQ‐8 is a valid and reliable measure of current depression for use in the general population (Kroenke et al., 2009). Items are scored on a Likert scale ranging from 0 (not at all) to 3 (nearly every day) and summed to create an overall score (range: 0–24), with higher scores indicating a greater severity of depression (*α* = 0.91). Total depression score was entered as a count variable in adjusted models.

### Analytic plan

We examined the relations between the individual PTSD symptom clusters (i.e., re‐experiencing, avoidance, negative cognitions and mood, and hyperarousal) and domains of hazardous drinking (i.e., consumption, dependence, and alcohol‐related problems) across five yearly assessments using generalized estimating equation (GEE) models with a negative binomial distribution for all AUDIT outcomes, a log‐link function, and an exchangeable correlation structure. Rate ratios (RRs) and 95% confidence intervals (CIs) are reported. Adjusted models controlled for the effects of anxiety and depression symptomatology, in addition to time. Each model first examined the main effects of the four PTSD symptom clusters on each domain of hazardous drinking. Subsequently, we tested two‐way interaction terms between each PTSD symptom cluster and biological sex (e.g., hyperarousal × sex) to examine whether associations differed for male versus female soldiers. Each interaction model also included the corresponding main effect of sex, as well as time, anxiety, and depression as covariates. Finally, we probed significant interactions and plotted the predictive margins of the corresponding domains of hazardous drinking by PTSD symptomatology and sex. Each PTSD symptom cluster (i.e., re‐experiencing, avoidance, negative cognitions and mood, and hyperarousal) was modeled separately in relation to each domain of hazardous drinking (i.e., consumption, dependence, and alcohol‐related problems) to reduce concerns about multicollinearity among clusters and to allow clearer interpretation of the unique association of each symptom domain with alcohol‐related outcomes. *p*‐Values less than 0.05 were considered statistically significant. All analyses were conducted in Stata/MP version 19.0 (College Station, TX).

## RESULTS

### Descriptive results

Among this sample of USAR/NG soldiers, the mean (standard deviation) total AUDIT score was 4.5 (4.2) and ranged from a low of 0 to a high of 36. Across all five waves, total AUDIT scores exceeded 8 (suggestive of harmful alcohol use) for 17.3% of male soldiers and 10.3% of female soldiers. The mean (standard deviation) AUDIT subscale scores were as follows: consumption = 3.3 (2.1), dependence = 0.4 (1.1), and alcohol‐related problems = 0.8 (1.8). Each subscale score ranged from a minimum of 0 to a maximum of 12. Male soldiers had a significantly higher mean total AUDIT score (*p* < 0.001), consumption subscale score (*p* < 0.001), and dependence subscale score (*p* = 0.040) than female soldiers. There were no differences in alcohol‐related problems subscale score by biological sex (*p* = 0.522). The overall PCL‐5 score ranged from 0 to 80, with a mean (standard deviation) of 9.9 (13.3). Mean (standard deviation) PTSD symptom cluster subscale scores were as follows: re‐experiencing (i.e., cluster B) = 2.0 (3.5), avoidance (i.e., cluster C) = 0.9 (1.6), negative cognitions and mood (i.e., cluster D) = 3.4 (5.0), and hyperarousal (i.e., cluster E) = 3.6 (4.5). There were no differences between male and female soldiers in mean overall PCL‐5 score (*p* = 0.342), re‐experiencing score (*p* = 0.257), or hyperarousal score (*p* = 0.250). Female soldiers had a significantly higher mean avoidance score (*p* = 0.023) and negative cognitions and mood score (*p* = 0.045) than male soldiers.

### Main effects of PTSD symptom clusters on domains of hazardous drinking

In main effects models controlling only for the effect of time, each PTSD symptom cluster (i.e., re‐experiencing, avoidance, negative cognitions and mood, and hyperarousal) was significantly associated with greater hazardous drinking across each domain (i.e., consumption, dependence, and alcohol‐related problems; Table 2). After controlling for the effects of anxiety and depression symptomatology, we found that re‐experiencing symptoms were not associated with any domains of hazardous drinking (*p*s >0.05). Greater avoidance symptoms (*p* < 0.001) and negative cognitions and mood (*p* < 0.05) were both associated with greater alcohol‐related problems. Greater symptoms of hyperarousal were associated with greater consumption (*p* < 0.05) and alcohol‐related problems (*p* < 0.01) over time. None of the symptom clusters were associated with dependence (*p*s >0.05).

**TABLE 2:** Main effects of PTSD symptom clusters on domains of hazardous drinking.

	Consumption		Dependence		Alcohol‐related problems	
	RR (95% CI)	aRR (95% CI)	RR (95% CI)	aRR (95% CI)	RR (95% CI)	aRR (95% CI)
Re‐experiencing (cluster B)	1.02 (1.01, 1.03)**	1.01 (0.99, 1.02)	1.10 (1.08, 1.13)***	1.01 (0.98, 1.05)	1.09 (1.07, 1.11)***	1.02 (0.99, 1.04)
Avoidance (cluster C)	1.05 (1.02, 1.07)**	1.02 (0.99, 1.05)	1.23 (1.17, 1.28)***	1.04 (0.97, 1.10)	1.22 (1.18, 1.26)***	1.09 (1.04, 1.14)***
Negative cognitions and mood (cluster D)	1.01 (1.01, 1.02)**	0.99 (0.99, 1.01)	1.08 (1.07, 1.10)***	1.02 (0.99, 1.04)	1.07 (1.06, 1.09)***	1.02 (1.01, 1.04)*
Hyperarousal (cluster E)	1.02 (1.01, 1.03)***	1.02 (1.01, 1.03)*	1.09 (1.08, 1.11)***	1.02 (0.99, 1.04)	1.09 (1.07, 1.10)***	1.03 (1.01, 1.06)**

*Note*: Adjusted models control for time, anxiety symptomatology, and depression symptomatology.

Abbreviations: aRR, adjusted rate ratio; CI, confidence interval; RR, rate ratio.

*p < 0.05; **p < 0.01; ***p < 0.001.

### Interaction effects of PTSD symptom clusters and sex on domains of hazardous drinking

**FIGURE 1:**
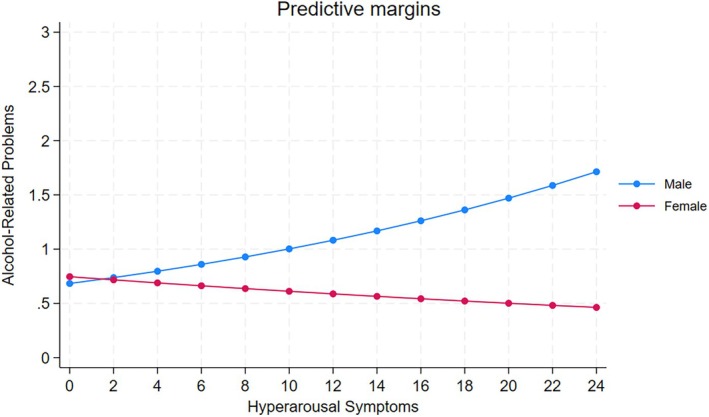
Predicted margins of alcohol‐related problems by hyperarousal symptoms and sex across five study waves, adjusted for time, anxiety, and depression. Greater hyperarousal symptoms were associated with greater alcohol‐related problems among male soldiers, but not female soldiers.

After adding an interaction term representing the cross‐products of the corresponding PTSD symptom cluster and biological sex to each fully adjusted model, we found a statistically significant interaction between symptoms of hyperarousal and biological sex on alcohol‐related problems (*p* < 0.01; Table 3). Greater symptoms of hyperarousal were associated with greater alcohol‐related problems among male soldiers, but not female soldiers (Figure 1). There were no other statistically significant interactions (*p* range: 0.30–0.78) between any of the PTSD symptom clusters and sex on any of the hazardous drinking domains.

**TABLE 3:** Interaction effects of PTSD symptom clusters and biological sex on domains of hazardous drinking.

	Consumption	Dependence	Alcohol‐related problems
	aRR (95% CI)	aRR (95% CI)	aRR (95% CI)
Re‐experiencing (cluster B) × biological sex	0.99 (0.96, 1.02)	0.97 (0.92, 1.03)	0.99 (0.95, 1.03)
Avoidance (cluster C) × biological sex	1.01 (0.95, 1.07)	0.98 (0.88, 1.11)	1.02 (0.94, 1.11)
Negative cognitions and mood (cluster D) × biological sex	1.00 (0.98, 1.02)	0.98 (0.94, 1.02)	0.99 (0.96, 1.01)
Hyperarousal (cluster E) × biological sex	0.99 (0.96, 1.01)	0.98 (0.93, 1.03)	0.94 (0.91, 0.98)**

*Note*: Male is the referent group; models control for the main effects of sex, time, anxiety symptomatology, and depression symptomatology.

Abbreviations: aRR, adjusted rate ratio; CI, confidence interval.

** *p* < 0.01.

## DISCUSSION

Reservists frequently navigate the dual demands of civilian and military life (Griffith, 2005, 2011; Vest, 2013), which can create challenges such as role conflict, limited access to health resources, and reduced social support from military peers (Hoopsick et al., 2018, 2019, 2021; Vest et al., 2023). These stressors may contribute to both alcohol use and PTSD symptoms, particularly during periods of transition such as post‐deployment reintegration or activation (Griffith, 2022). This study provides important insight into the associations between distinct PTSD symptom clusters and domains of hazardous drinking over time among USAR/NG soldiers, a population at elevated risk for both trauma‐related pathology and alcohol misuse. By disaggregating PTSD into its symptom clusters and examining their unique associations with specific domains of hazardous drinking over time, we identified patterns that are obscured when examining only total symptom scores or aggregated drinking outcomes. In this sample of 485 USAR/NG soldiers, only certain PTSD symptoms were associated with hazardous drinking. Our adjusted main effects models suggest that the relation between PTSD and hazardous drinking may manifest primarily in alcohol‐related problems, with greater PTSD symptoms of avoidance, negative cognitions and mood, and hyperarousal being associated with greater alcohol‐related problems. Additionally, the association between PTSD and hazardous drinking appears to be driven more by hyperarousal than by other symptoms of PTSD; greater hyperarousal was associated with both greater alcohol consumption and greater alcohol‐related problems.

The observed associations involving avoidance and negative cognitions and mood symptoms may reflect distinct pathways linking PTSD to hazardous drinking. Avoidance symptoms could promote alcohol use as a strategy to disengage from trauma‐related thoughts or emotions, whereas negative cognitions and mood may heighten vulnerability to using alcohol as a maladaptive coping mechanism to manage distress or social isolation. These mechanisms align with prior work suggesting that trauma‐related avoidance and negative affect both contribute to alcohol misuse through emotion regulation deficits and negative reinforcement processes (Baker et al., 2004; Jakupcak et al., 2010).

Interestingly, none of the individual PTSD symptom clusters were associated with alcohol dependence in the current study, suggesting these symptom dimensions may be more closely related to consumption and alcohol‐related problems than to physiological dependence. (e.g., not being able to stop drinking after starting a drinking episode, failing to do what is normally expected as a result of drinking, needing to drink first thing in the morning to get going after a heavy drinking session). One possible explanation is that dependence, as measured by the AUDIT, captures more chronic or severe patterns of use that may not be as sensitive to fluctuations in PTSD symptoms over time. It is also possible that the relatively low levels of hazardous drinking in this community‐based, non‐clinical sample limited our ability to detect significant effects. It could also be possible that the association between overall PTSD symptom severity and alcohol dependence observed in other studies might be a function of total symptom burden across all the clusters rather than a specific set of PTSD symptoms driving this association. Additionally, the co‐morbidity of alcohol dependence among people with PTSD may, at least in part, be due to shared genetic etiology (Sartor et al., 2011; Sheerin et al., 2020; Xian et al., 2000). These findings highlight the need for further longitudinal studies with longer follow‐up durations or clinical interviews to better capture potential pathways from PTSD symptoms to physiological dependence.

Notably, the current study revealed a significant interaction between symptoms of hyperarousal and biological sex on alcohol‐related problems, such that worse symptoms of hyperarousal were associated with greater alcohol‐related problems, but only for male soldiers. Several mechanisms may explain this sex‐specific effect. First, men may be more likely than women to use alcohol as a primary coping strategy for managing psychological distress (Keyes et al., 2012), consistent with gender role socialization theories (Schulte et al., 2009). This may reflect broader gendered patterns in coping and substance use, where men are socialized to externalize distress and use substances to self‐regulate physiological arousal, whereas women may internalize distress or seek social support. Second, sex‐based differences in stress reactivity and neuroendocrine functioning may contribute to differential reinforcement effects of alcohol, whereby men may experience more immediate relief from hyperarousal symptoms through drinking than women (Becker & Koob, 2016; Chaplin et al., 2008; Jezová et al., 1996). Additionally, military culture often reinforces masculine norms around stoicism and drinking (Ames et al., 2007; Fox & Pease, 2012; Hinojosa, 2010; Meadows et al., 2023), which may further reinforce alcohol use as an acceptable or expected method of emotion regulation among male service members. These findings highlight the need for sex‐specific approaches to screening and intervention, particularly strategies that address hyperarousal symptoms and promote adaptive coping mechanisms among men at risk for hazardous drinking.

The descriptive findings from this sample of USAR/NG soldiers also provide important context for understanding patterns of alcohol use and PTSD symptomatology within this population. Overall, the mean AUDIT score of 4.5 indicates generally low levels of hazardous drinking. However, a wide range in scores suggests that a subset of soldiers is at elevated risk for alcohol‐related problems. The highest mean scores were observed on the consumption subscale, suggesting that frequency and quantity of drinking, rather than dependence or consequences, are the most prevalent concerns. Notably, male soldiers reported significantly higher total AUDIT scores and greater levels of alcohol consumption and dependence compared to female soldiers, underscoring sex‐based differences in drinking behaviors that align with existing literature on military populations. While no sex differences were observed in total PTSD symptom burden, female soldiers reported significantly higher scores on avoidance and negative cognitions and mood, which may reflect sex‐specific manifestations of trauma response. These patterns highlight the importance of examining both overall symptom severity and specific symptom dimensions, as well as considering sex differences in mental health and substance use research within military samples.

The findings of this longitudinal study also provide support for both the self‐medication hypothesis and the negative reinforcement model of alcohol use among military service members with PTSD, particularly among male soldiers. Specifically, we found that hyperarousal symptoms were associated with increased alcohol consumption and alcohol‐related problems. According to the self‐medication hypothesis (Khantzian, 1997), individuals may turn to alcohol to alleviate these distressing symptoms, using it as a form of coping or sedation. This interpretation is further supported by the negative reinforcement model (Baker et al., 2004), which posits that substance use is maintained over time because it provides short‐term relief from negative emotional or physiological states. The consistent associations between hyperarousal symptoms and domains of hazardous drinking observed in the current study support the idea that alcohol use may be particularly appealing to some service members to reduce autonomic arousal and promote short‐term emotional regulation. These findings underscore the importance of targeting hyperarousal symptoms and maladaptive coping strategies in interventions designed to prevent or reduce hazardous drinking in trauma‐exposed populations.

### Limitations and strengths

This study is not without limitations. As with all survey‐based research, there is a potential for response bias with self‐reported data. However, given the use of validated clinical measures and the use of a confidential survey, the risk of social desirability bias was low. Additionally, all participants were either married or living as married at baseline. This may limit generalizability, but national data indicate that the majority of service members are married (Department of Defense, 2020). Participants were also recruited from a single US state, which may also affect the external validity of our findings. However, these units were diverse in terms of soldiers' military occupational specialties and included combat, engineer, medical, logistics, and support roles. The characteristics of our sample are also representative of USAR/NG soldiers in terms of age, race, and sex distribution (Department of Defense, 2021). Moreover, additional strengths include our longitudinal design, a focus on an often understudied and high‐risk population, and the disaggregation of both PTSD symptom clusters and domains of hazardous drinking, all of which add to the contribution of the current study to the extant literature.

Although our findings highlight potential pathways linking PTSD symptom clusters to hazardous drinking, it is important to interpret these results in light of the sample characteristics. Participants in this study reported relatively low levels of PTSD symptoms and hazardous drinking overall, which limits generalizability to clinical or treatment‐seeking populations. Consequently, the present findings should be viewed as reflecting patterns of association among symptom domains rather than evidence of causal mechanisms or treatment implications.

## CONCLUSION AND FUTURE DIRECTIONS

Our findings suggest that symptoms of avoidance, negative cognitions and mood, and hyperarousal are especially relevant to the development of alcohol‐related problems, with hyperarousal also contributing to higher levels of alcohol consumption. These results reflect models adjusted for anxiety and depression symptomatology, and some associations observed in unadjusted models (e.g., re‐experiencing symptoms and alcohol dependence) were no longer significant after adjustment, highlighting the importance of accounting for comorbid internalizing symptoms. Importantly, none of the PTSD symptom clusters were associated with alcohol dependence over time, highlighting the possibility that specific PTSD features contribute more to problematic drinking behaviors and consequences than to physiological dependence.

Ultimately, this study contributes to a growing body of evidence suggesting that a more nuanced, symptom‐specific, and sex‐sensitive approach may be essential to developing effective prevention and intervention strategies for addressing co‐occurring PTSD and hazardous drinking in military populations, especially reservists who may face unique challenges in accessing care and support. Findings contribute to a broader understanding of the psychopathological processes through which PTSD symptoms, particularly avoidance, negative cognitions and mood, and hyperarousal, may shape alcohol‐related behaviors, but additional work in clinical samples is needed to determine whether these associations inform intervention development.

Future research should explore the mediating or moderating roles of other psychological factors (e.g., emotion regulation, coping motives) in the relation between PTSD symptom clusters and hazardous drinking. Additionally, studies using ecological momentary assessment or other real‐time data collection methods could provide more nuanced insights into how specific PTSD symptoms relate to alcohol use behaviors on a day‐to‐day basis. Such approaches may offer critical insight into the temporal dynamics of symptom‐triggered drinking episodes and support the development of clinical interventions.

Collectively, the findings from the current study advance understanding of how distinct PTSD symptom clusters may contribute to hazardous drinking behaviors in a high‐risk yet understudied military subpopulation. By clarifying the nuanced, symptom‐specific, and sex‐sensitive pathways linking PTSD and alcohol use, this work contributes to the broader goal of improving prevention, screening, and conceptual models of comorbid trauma and substance use among service members and veterans.

## CONFLICT OF INTEREST STATEMENT

The authors have no conflicts of interest to decalre.

## Data Availability

The data that support the findings of this study are available on request from the corresponding author. The data are not publicly available due to privacy or ethical restrictions.
